# ESR and NMR studies provide evidence that phosphatidyl glycerol specifically interacts with poxvirus membranes

**DOI:** 10.1186/1743-422X-7-379

**Published:** 2010-12-31

**Authors:** Jean-Claude Debouzy, David Crouzier, Anne-Laure Favier, Julien Perino

**Affiliations:** 1 Unité de biophysique cellulaire et moléculaire, CRSSA-IRBA, 24 avenue des maquis du Grésivaudan, 38702 La Tronche cedex France; 2 Laboratoire de virologie, département de microbiologie. CRSSA-IRBA, 24 avenue des maquis du Grésivaudan, 38702 La Tronche cedex France

## Abstract

**Background:**

The lung would be the first organ targeted in case of the use of Variola virus (the causative agent of smallpox) as a bioweapon. Pulmonary surfactant composed of lipids (90%) and proteins (10%) is considered the major physiological barrier against airborne pathogens. The principle phospholipid components of lung surfactant were examined in an *in vitro *model to characterize their interactions with VACV, a surrogate for variola virus. One of them, Dipalmitoyl phosphatidylglycerol (DPPG), was recently shown to inhibit VACV cell infection.

**Results:**

The interactions of poxvirus particles from the Western Reserve strain (VACV-WR) and the Lister strain (VACV-List) with model membranes for pulmonary surfactant phospholipids, in particular DPPG, were studied by Electron Spin Resonance (ESR) and proton Nuclear Magnetic Resonance (^1^H-NMR). ESR experiments showed that DPPG exhibits specific interactions with both viruses, while NMR experiments allowed us to deduce its stoichiometry and to propose a model for the mechanism of interaction at the molecular level.

**Conclusions:**

These results confirm the ability of DPPG to strongly bind to VACV and suggest that similar interactions occur with variola virus. Similar studies of the interactions between lipids and other airborne pathogens are warranted.

## Background

Membrane contacts occur at the very early steps of pulmonary viral infection [1]. This is especially important under circumstances where aerosol dispersion of viruses occurs readily [2] and an aerosol respiratory contamination would be an easy way for a biological agent to cause massive casualties. One of the initial and essential steps in a pulmonary infection is the crossing of the surfactant barrier separating the respiratory lumen from cells lining alveoli. The interactions of vaccinia virus (VACV, a surrogate model of variola) with surfactant phospholipid components, is the focus of the present work. Pulmonary surfactant (PS) is a complex mixture of lipids (90%) and proteins (10%), participating in reducing surface tension at the air-liquid interface and in protecting the lung against pathogens as part of the innate immune system [3-6]. The abundance and physiological importance of several phospholipid species (ie Phosphatidylcholine, PC; Dipalmitoyl phosphatidylcholine, DPPC; Dipalmitoyl phosphatidylglycerol, DPPG) led us to select several of them in the study of virus interactions with surfactant [3,4,7]. To date, only a few studies have described the role of surfactant phospholipids in virus entry. In the case of adenoviruses, the role of DPPC contained in lung surfactant or expressed by lung cells was found to increase the penetration of a respiratory adenovirus without involving any specific receptors [8]. The specific interaction of an enteric adenovirus strain with different phospholipids contained in the gastrointestinal surfactant has also been characterized [9]. Considering the importance of phospholipids in lung surfactant and the hypothesis that specific virus-phospholipid interactions may occur we were interested in gaining an understanding of VACV entry into alveolar epithelium. Recently, we showed that DPPG interacts with VACV and that DPPG incorporated in Small Unilamellar Vesicles (SUV-DPPG) inhibits VACV cell infection, unlike other phospholipids tested [10]. In this study, we first focused on the major components of phospholipid lung surfactant and two viral strains were selected (the virulent lethal mouse neurotropic Western Reserve strain (VACV-WR) [11] and the Lister strain (VACV-List), previously used in Europe as a smallpox vaccine). In this *in vitro *study, electron spin resonance (ESR) and nuclear magnetic resonance (NMR) methods were used to identify and draw mechanistic data of virus phospholipid interactions, using small unilamellar vesicles (SUV), previously established as membrane models [12].

## Methods

### Virus preparation and inactivation

The vaccinia virus Western Reserve strain (VACV-WR), obtained from the ATCC (ATCC VR-119), and the first-generation Lister smallpox vaccine (VACV-List), provided by the French health authorities, were produced in BHK-21 cells and titrated in Vero cells. In order to use these viruses for NMR experiments, solvents were replaced by deuterated solvents. Viruses purified in water based solvents were diluted in deuterated PBS and then purified using deuterated sucrose gradients [13].

For safety reasons, viruses were inactivated for some experiments using a previously described protocol [14]. Briefly, 100 μL virus was incubated with 1 μL Psoralen (Sigma, 1 mg/mL in Deuterated DMSO) and exposed for 1 hour to UV light (365 nm) in a 48 well tissue culture plate. For the samples dedicated to NMR experiments, the same preparation was used except that all solvents (water, DMSO….) were deuterated to avoid spectrum saturation related to an excessive contribution of the solvent resonances. The final amount of virus was 4.5.10^9 ^PFU in a 500 μL sample.

### Small unilamellar vesicles (SUV)

Freeze-dried phospholipids were dissolved in chloroform at the desired molar concentration. Unsaturated phospholipids (DPPC or DPPG) were added to a 2 mM PC solution in a 30% final ratio. The mixture was dried overnight under vacuum. The lipid film was hydrated with water and subjected to water bath sonication for 2 hours at different temperatures depending on the fusion temperature of the lipids present in the mixture. SUV formation was ascertained by the observation of a ^1^H-NMR classical spectrum, with a typical linewidth of chain terminal methyl groups of 15 Hz or less [15]. For the NMR experiments recorded in the presence of dipalmytoyl phosphatidylglycerol (DPPG), the lipid concentration of the stock solution was 2.7 mM in D_2_O.

### ESR Experiments

SUV/virus interactions were assessed by Electron Spin Resonance (ESR) spin labeling experiments. Inactivated virus (5 μL, 9 x10^9^/mL) was labeled with the 5-nitroxide stearate (5NS) probe (Sigma France), (5 min incubation at 37°C). This probe is composed by a fatty acid (C16) and a stabilized free radical. The probe self incorporates into membranes and provides information of label motional freedom in the system. Then the specific SUV solution (50 μL at 2 mM) was added to the mix and incubated for 90 minutes at 37°C. The beginning of the kinetics was triggered by addition of C Vitamin (L-ascorbic acid, 15 μL, 0.2 M). C Vitamin is a well known free radical scavenger. The decrease of the ESR signal could be linked to the accessibility of the probe to the C Vitamin, so a rapid decrease implies very few SUV/Virus interactions.

Five minutes after C vitamin addition, the kinetics were recorded on an ESP 380 Brucker apparatus. Spectra were acquired in time sweep mode using static field, determined at the central line maximum amplitude of the T0 spectrumunder in EPR continuous wave mode (3428 G). The instrument parameters were microwave power at 10 mW, modulation frequency at 100 kHz, modulation amplitude at 2.53 G and receiver gain at 6.30 x10^4^. Each sample was scanned 3 times under a controlled temperature (300 K) with the following acquisition parameters: Time constant 163.84 ms, conversion time 163.84 ms. All kinetics were recorded for 671 seconds and all experiments were performed in triplicate. For most ESR curve fitting, an exponential fit was used and considered as correct for values of R exceeding 0.95. In the remaining cases, more complex functions had to be used and the fit on 671 data points was directly validated by a χ^2 ^test when the calculated value did not exceed 4 [16].

### NMR Experiments

^1^H-NMR spectra were recorded at 295 K on an AM400 Bruker spectrometer at 400 MHz (9.4 T), using a presaturation sequence for water resonance suppression. The spectral width was 6000 Hz (15 ppm) recorded on 32 K data acquisition points with a recycling delay of 1 s. Each spectrum was recorded using 80,000 scans.

## Results and discussion

### ESR experiments: specificity of DPPG-VACV interactions

**Figure 1:**
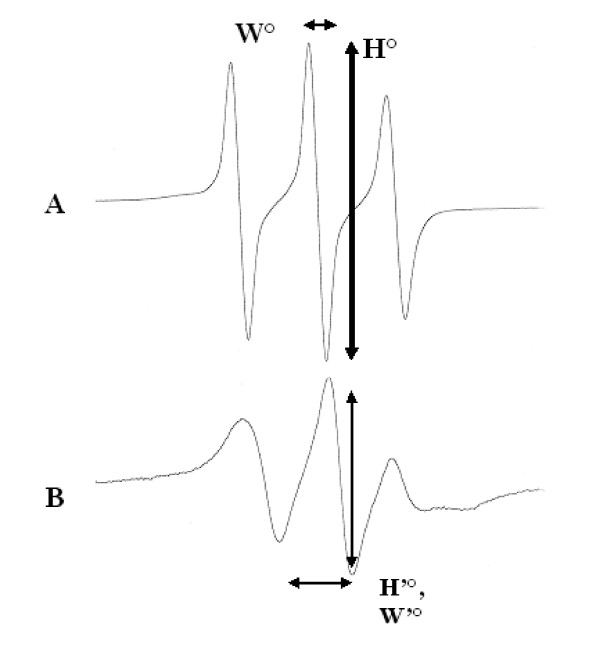
**Typical ESR spectra**. ESR spectra was recorded (295 K) under free motion conditions (A) and in the presence of motional limitation by interactions with lecithin vesicles (B). Respective linewidth (W° W'°) and peak to peak height (H° H'°) are labeled with arrows.

A typical ESR spectrum of the 5-nitroxide stearate (5NS) label is recorded under free motion conditions (Figure 1A) and in the presence of small unilamellar vesicles (SUV), composed of PC (SUV-PC) (Figure 1B). Here it is noteworthy to recall some basic concepts required to draw any interpretation of such spectra. Each nitroxide group of the 5NS label bears a single NO° free radical, chemically stabilized by the surrounding methyl groups of the nitroxide. Under the magnetic resonance conditions (i.e. a 9.71 GHz radiofrequency, a magnetic screening on a 100 Gauss window), and due to hyperfine spin coupling, this group gives rise to 3 distinct lines. At this step, the spectral information used in this paper are:

- Spectral magnitude/intensity which at a first approximation is directly related to the number of spins present in the sample, which itself is related to the 5NS concentration.

- The magnitude of the resonance is in fact also dependent on the line width (W°). For a given spin system, the product of W° by the peak height (H°) is a constant. This means that any line broadening will result in H° intensity reduction (e.g. H'° and W'° in Figure 1B).

- W°, theoretically close to zero in ideal systems (free motion, no interaction or inhomogeneity) will broaden under two main circumstances, motional restriction/relaxation and exchange. The former is illustrated in Figure 1A, where resolved lines are detected on the spectrum of free 5NS while broader and less intense lines are detected on the motionally restricted 5NS embedded in the membrane (Figure 1B). The latter observation results from the simultaneous existence of spins differing by their motional freedom. If the exchange is slow, the two components give rise to separate contributions (intermediate and fast exchanging systems only allow the detection of an average value of H° and W°) from those of the two components.

- The magnitude of a spectrum is difficult to determine so its peak height measurement is generally used (in fact the peak to peak height, labeled H°). Any spin label reduction or destruction results in a proportional diminution of this value if the line width is kept constant. Thus, the time course of the central line height was recorded in the following experiments while controlling the line width before any interpretation of the data.

### Time course of 5NS

#### Phospholipid systems (SUV)

**Figure 2:**
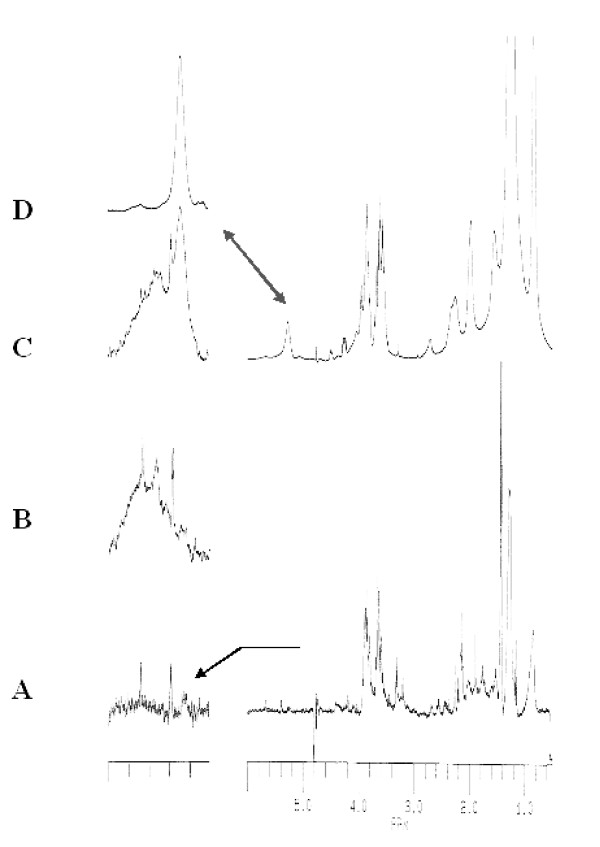
**^1^H-NMR spectra**. ^1^H-NMR spectra of A) virus suspension in D2O, 295 K (2.10^9^/μL), with expanded plots of the 5-6 ppm region, left. B) Same conditions as in A after addition of 50 μL DPPG, 2.7 mM, D2O. C) same as B), with 100 μL total amount of DPPG. D) spectrum of pure SUV-DPPG with expanded 5-6 ppm region presented in the left column.

**Figure 3:**
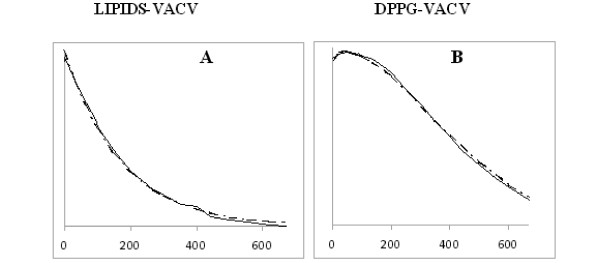
**Peak to peak height evolution**. Peak to peak height evolution measured on the central line of spectra as in Figure 1 after (15 μL, 0.2 M) ascorbate addition for DPPC VACV-List sytems (A), and DPPG + VACV-List systems (B).

After incubation of the spin label with SUV alone, a typical spectrum of 5NS in the membrane was observed, providing the reference intensity and linewitdh. Also, the absence of a resolved line ensured that the entire label was incorporated in the bilayer and that no free spin label remained in the bulk. The addition of ascorbate (Vitamin C, VitC) results in the reduction of the nitroxide, which can be visualized by a reduction in intensity. As the penetration is progressive and intra-membrane motion of the label occurs, the time course curve presented in Figure 2A is easily fitted by an exponential function, as presented in Figure 3A and the associated table. This time dependence was found for all of the pure SUV systems with time constants of the same order of magnitude.

#### DPPC, PC, Sulfatide, and VACVs

Coincubation of viruses with SUV and 5NS prior to VitC addition resulted in spectra similar to those recorded in the presence of SUV-5NS alone (peak height and line width were very close). Furthermore, the addition of ascorbate induced a similar time dependence of peak intensity reduction as testified by similar time constants of the exponential curves (table 1).

**Table 1:** Numerical characteristics of the fits shown on the top traces. General formulae were H(t) = H°. exp(-t/τ)* cos(a.t + ϕ)

Phospholipid	Curve	1/Tau	Phase	Cosinus
**PC**	monoexponential	280	-	-

**PC + VACV-WR**	monoexponential	180	-	-

**PC + VACV-List**	monoexponential	227	-	-

**Sulfatide**	monoexponential	250	-	-

**Sulfatide + VACV-WR**	monoexponential	245	-	-

**Sulfatide + VACV-List**	monoexponential	300		-

**DPPC**	monoexponential	350	-	-

**DPPC + VACV-WR**	monoexponential	170	-	-

**DPPC + VACV-List**	monoexponential	180	-	-

**DPPG**	monoexponential	220	-	-

**DPPG + VACV-WR**	cosine* exponential	430	0	2.9E-3*t

**DPPG + VACV-List**	cosine* exponential	286	-0,8	2.7E-3*t

#### DPPG and VACVs

The same observation was made when SUV-DPPG were used without any virus. In contrast, co-incubation of SUV with 5NS labeled virus resulted in a distinct time dependence. An initial increase in signal intensity relative to the reference intensity was noted when SUV were added suggesting that either 5NS was less motionally restricted and/or that exchanges had occurred. This was supported by a initial value for W° of 4.7 Gauss, which increased to 6 Gauss at the maximum of the curve. At this time point VitC was present in the bulk. Longer time recordings led to a monoexponential decrease of the signal intensity with similar time constants as those observed for the other samples. Finally, the entire recording could not be fitted by a single exponential but required a diphased cosine component in addition to the exponential decrease (see Figure 3B and table 1). The time course recordings for samples with VACV-List or VACV-WR were similar even if the initial rise was more marked and the cosine parameters different when the VACV-List variant was used.

### ^1^H-NMR: mechanism of DPPG-VACV interaction

#### Spectrum of poxvirus, D_2_O

The spectrum displayed in Figure 2 (line A, right) appears as broad overlapping lines and relatively resolved resonances that preclude any clearcut interpretation. On such a spectrum, only the mobile superficial groups produce resolved lines, while strongly immobilized and/or embedded groups are not detected or produce the extremely broad contributions. A special region of interest is presented on the left trace of Figure 2A. Except for three multiplets at 5.2, 5.4 and 5.55 ppm, the 5-6ppm is free of any broad contribution.

#### DPPG addition

Increasing amounts of SUV-DPPG (2.7 mM, D_2_O) were added and similar spectra were recorded as for pure viruses. Up to 50 μL, no clear spectral modification was observed. Higher amounts allowed the detection of a very broad line of 250 Hz width whose magnitude increased with SUV concentration (Figure 2B).

Observation of the spectrum of pure SUV-DPPG (Figure 2D, expanded on the left column) showed that the only resonance in this spectral region is attributed to the glycerol methynic proton of the headgroup. However, such a resonance is located at 5.3 ppm and has only a 20 Hz linewidth, in agreement with the mobility and small size of SUV (10 nm). Such a feature could be related to lipid immobilization which may increase the linewidth and a change in the magnetic shielding of CH proton (e.g. due to interactions with the lipidic environment).

**Figure 4:**
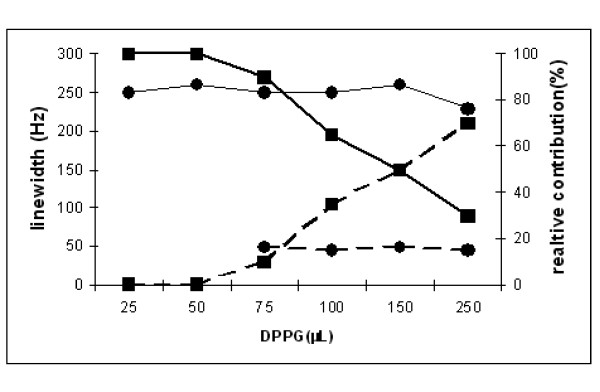
**Plots of the linewidth (black circle, in Hz) and of the relative contributions (black square, in %) of the spectral components identified in the 5-6 ppm region**.

Further evidence was obtained when supplementary amounts of DPPG were added. With 75 μL SUV, a third line was detected, identical to that of pure SUV with DPPG (compare Figure 2C with respect to 2D), whose magnitude increased with increasing amounts of DPPG. On the other hand, the broad contribution at 5.5 ppm remained unaffected. The SUV concentration dependence of the different contributions and linewidths are shown in Figure 4. This clearly suggests that ***i) ***the broad line observed at "the low concentration" results from DPPG-VACV interactions ***ii) ***that a saturation occurred for higher concentrations, leading to a major contribution of free SUV. This hypothesis is also supported by the markedly constant values of the linewidths of the two broad contributions (*i.e *250 and 45 Hz,). No other information could be drawn from spectral subtractions between spectra of pure and DPPG-associated viruses in order to distinguish between overlapping of virus and lipid resonances. However, the different resonances observed in the 5-6 ppm region undoubtedly ensure that this group and consequently the polar head group of DPPG is involved in DPPG-VACV interactions.

### Stoichiometry estimation

Using the starting concentration roughly extrapoled from the data in Figure 4 at 60 μL, an attempt to estimate the stoichiometry of the virus DPPG interactions was undertaken. Given the average cylindrical structure of the virus, considered as having a mean length of 350 nm and a radius of 175 nm [17], the total surface of the virus was estimated to be around **Sv **= ***0.47 μm***^***2***^***, (4.7 10***^***5***^***nm***^***2***^***). ***Considering an estimation of the surface of a phospholipid around *Sp ****≈ ****0.6-0.7 nm*^*2 *^(derived from the lecithin model), one can deduce for a phospholipid concentration of *C = 2.7 mM *in D_2_O, ***V ****= 60 μ*L added to the sample, the number of DPPG molecules added at this concentration:

(1) n=N*C*Sp*V

with N = 6.02 10^23^, the Avogadro number

(2) $$\text{n}\approx 10^{15}\;\text{molecules}$$

This leads to a total surface ST of:

(3) ST=Sp*n,

(4) $$\text{ST}\approx 7\;10^{14}\;{\text{nm}}^{2}$$

This allows an approximation of the number **Ne **of DPPG molecules involved in the interaction with the virus, assuming the absence of supramolecular structures remaining after SUV interactions:

(5) $$\text{Ne}=\text{ST}/\text{Sv}\approx 1.5\;10^{9}\;\text{DPPG}/\text{virus}$$

The result of this calculation appears somewhat unrealistic. Another way to calculate the stoichiometry is to consider the individual surface of a given headgroup on one hand (Sp), and the surface of the virus on the other hand (Sv). The maximum number of adducted headgroups would then be **Na**:

(6) $$\text{Na}=\text{Sv}/\text{Sp}\approx 8\;10^{5}$$

Finally, SUV vesicles are well known to be very stable and dissymmetric structures, containing about **R ***= 2-3000 *phospholipids per vesicle (1/3 in the internal layer, 2/3 in the external layer). This feature, correlated with the discrepancy between the two calculations given in equations (5) and (6), indicates that supramolecular assemblies are still present even below the saturation, since the Na*R product is in the same range as the **Ne**, ie 10^9^, with a difference of by a factor 3-4.

#### Correlation with ESR results

If one recalls the cosine*exponential dependence of the nitroxide signal reduction in the presence of ascorbate, it appears that the exponential decrease results from a well known mechanism of nitroxide reduction in SUV, since the membrane embedded spin label exhibits local motions and exchanges with the surface, making it accessible to reduction (and *de facto *to signal intensity diminution). Moreover, the cosine function and more generally the circular function dependence, may reveal exchange processes [18] occurring between different states. Three systems are in presence: the vesicles, the aqueous system where ascorbate is soluble and the virus itself. Here, an initial increase of the signal precludes a direct access of nitroxide to ascorbate that would lead to immediate set up of the intensity reduction.

Direct SUV-VACV adducts all appear favorable to intra membrane exchanges of the label without any requirement for contact with the ascorbate-containing bulk. This motion would also result in ESR line narrowing, i.e. for a constant amount of spin label (integral of the peak) an increase of the measured peak-height as observed in the initial part of the DPPG-VACV ESR curves should occur.

## Conclusions

When considering the respiratory route of contamination by VACV, the crossing of the surfactant barrier separating the respiratory lumen from cells lining alveoli is crucial [4]. The role of phosphatidylglycerol in the physico-chemical properties of surfactant was identified quite a while ago [19]. This study presents a systematic screening of the phospholipid-VACV interactions by the ESR method. This led us to identify an authentic interaction of glycerol bearing phospholipids with the virus and to use 1H-NMR to obtain more precise information about this interaction. We propose that DPPG interacts with the virus surface without requiring an intermediate aqueous phase but rather a close contact of supramolecular assemblies, such as SUV, potentially allowing exchanges of small molecules embedded in the membranes, as suggested by the ESR spin label. This implies a mechanism that could allow virus to overcome the alveolo-capillary barrier.

## Competing interests

The authors declare that they have no competing interests.

## Authors' contributions

JCD designed the study, JP and ALF were involved in the study design. JP prepared purified virus. DC and JCD performed acquisition of data. JCD analyzed the data. ALF, JP and JCD wrote the draft of the manuscript. All authors read and approved the final manuscript.
